# Prevention of musculoskeletal disorders in workers: classification and health surveillance – statements of the Scientific Committee on Musculoskeletal Disorders of the International Commission on Occupational Health

**DOI:** 10.1186/1471-2474-13-109

**Published:** 2012-06-21

**Authors:** Mats Hagberg, Francesco Saverio Violante, Roberta Bonfiglioli, Alexis Descatha, Judith Gold, Brad Evanoff, Judith K Sluiter

**Affiliations:** 1 Department of Public Health and Community Medicine, University of Gothenburg (UGOT), Box 414, SE 405 30, Gothenburg, Sweden; 2 Occupational Health Unit, University of Bologna Sant’Orsola Malpighi Hospital via Palagi, 9, 40138, Bologna, Italy; 3 Occupational Health Department/INSERM U1018-UVSQ Poincaré Teaching Hospital AP-HP, Garches, France; 4 Department of Public Health, Temple University, 1301 Cecil B. Moore Avenue, Ritter Annex, Philadelphia, PA 19122, USA; 5 Division of General Medical Sciences, Washington University School of Medicine, Campus, Box 8005, 660 S, Euclid Ave., St. Louis, MO 63130, USA; 6 Academic Medical Centre, Department: Coronel Institute of Occupational Health, Meibergdreef 9, 1105 AZ, Amsterdam, The Netherlands

**Keywords:** Occupation, Epidemiology, Prevention, Aetiology, Expert opinion, Occupational health, Public health, Rheumatology, Rehabilitation, Orthopaedics

## Abstract

The underlying purpose of this commentary and position paper is to achieve evidence-based recommendations on prevention of work-related musculoskeletal disorders (MSDs). Such prevention can take different forms (primary, secondary and tertiary), occur at different levels (i.e. in a clinical setting, at the workplace, at national level) and involve several types of activities. Members of the Scientific Committee (SC) on MSDs of the International Commission on Occupational Health (ICOH) and other interested scientists and members of the public recently discussed the scientific and clinical future of prevention of (work-related) MSDs during five round-table sessions at two ICOH conferences (in Cape Town, South Africa, in 2009, and in Angers, France, in 2010). Approximately 50 researchers participated in each of the sessions. More specifically, the sessions aimed to discuss new developments since 1996 in measures and classification systems used both in research and in practice, and agree on future needs in the field.

The discussion focused on three questions: At what degree of severity does musculoskeletal ill health, and do health problems related to MSDs, in an individual worker or in a group of workers justify preventive action in occupational health? What reliable and valid instruments do we have in research to distinguish ‘normal musculoskeletal symptoms’ from ‘serious musculoskeletal symptoms’ in workers? What measures or classification system of musculoskeletal health will we need in the near future to address musculoskeletal health and related work ability?

Four new, agreed-upon statements were extrapolated from the discussions: 1. Musculoskeletal discomfort that is at risk of worsening with work activities, and that affects work ability or quality of life, needs to be identified. 2. We need to know our options of actions before identifying workers at risk (providing evidence-based medicine and applying the principle of best practice). 3. Classification systems and measures must include aspects such as the severity, frequency, and intensity of pain, as well as measures of impairment of functioning, which can help in prevention, treatment and prognosis. 4. We need to be aware of economic and/or socio-cultural consequences of classification systems and measures.

## Background

In 1996, the Scientific Committee (SC) on Musculoskeletal Disorders (MSDs) of the International Commission on Occupational Health (ICOH) (http://www.icohweb.org) published a position paper [1] on work-related risk factors and prevention of MSDs in workers. The conclusion back then was that international organizations should work to develop standards, common classifications, and uniform terminologies related to MSDs. In addition, surveillance systems should be further developed nationally, based on the International Labour Organization (ILO)’s definition of ‘surveillance’ [2] (http://www.ilo.org):

Workers' health surveillance is a generic term which covers procedures and investigations to assess workers’ health in order to detect and identify any abnormality. The results of surveillance should be used to protect and promote the health of the individual, collective health at the workplace, and the health of the exposed working population. Health assessment procedures may include, but are not limited to, medical examinations, biological monitoring, radiological examinations, questionnaires or a review of health records.

Since the first position paper and especially in the first decade of this century, the literature on surveillance of MSDs in workers presents ongoing discussions concerning both the definitions and the relevance of measured outcomes in musculoskeletal occupational medicine [3-10]. This literature aimed to facilitate a more uniform collection, recording and reporting of information about (work-related) MSDs across countries by providing evidence- or consensus-based case definitions and criteria for identifying and classifying them. Noticeably, these publications have been the result of the collaborative effort of several groups of experts, and provide an invaluable contribution to harmonize the scientific approaches to prevention of (work-related) MSDs. In the literature reviews of diagnostic criteria, the definitions and official statistics were found to be rarely comparable across countries, for several good reasons. However, the consensus-based publications of definitions and criteria should facilitate the practical work of those involved in this field, as shown in later studies (see, e.g., [11-13].

Despite the impressive number of studies on (work-related) low back and upper extremity MSDs in worker populations, considerable uncertainty and even controversy still exists about the extent and aetiology of these problems, the contribution of work and non-work risk factors to their development and resolution, the criteria used to diagnose them, the outcomes of various treatment methods and, of utmost importance, the appropriate strategies for intervention and prevention.

One and a half decades and another several reviews after the 1996 paper of the ICOH’s SC on MSDs, we have to acknowledge that agreed-upon knowledge is still lacking about meaningful case definitions and about possible underlying mechanisms for the development of non-specific musculoskeletal symptoms in particular and (chronic) pain in general. It remains difficult to compare the results of different epidemiological studies, surveillance systems, and registration databases (e.g. [14]. This difficulty hampers efforts to assess and compare the magnitude and nature of work-related MSDs within and across different countries, geographic areas, industries, workplaces, and occupational groups over time. Similarly, it impedes the ability to assess the effectiveness of different types of medical and workplace interventions. Scientific progress in advancing our understanding of the abovementioned problems is still being hampered by methodological and practical challenges associated with epidemiological research on work-related MSDs (e.g. [15].

Having said this, our scientific knowledge should be designed to be used in practice, primarily for occupational health physicians who provide care for individual workers with health problems and who provide occupational health services to workers and employers in different companies. The recognition of work-related disease and injury often begins in the physician’s office, once patients decide to seek help for their symptoms, complaints or functional limitations. Clearly, the clinical process is dynamic and physicians use their best medical judgment in making a diagnosis and decide on what action is necessary. Sometimes, information about work-related MSDs is solicited in a more active way to help identify existing or potential problems and risks in certain occupational groups or in particular workplaces. This type of action can be initiated by occupational health professionals who provide prevention-oriented services to companies and groups of workers, often at the request of employers or workers. Health or labour authorities may engage in or require occupational health surveillance for high-risk groups or when alerted to possible problems through other means.

## Main text

The underlying purpose of this article and position paper is to achieve evidence-based recommendations on prevention of work-related MSDs. Such prevention can take different forms (primary, secondary and tertiary), occur at different levels (i.e. in a clinical setting, at the workplace, at national level) and involve several types of activities.

Members of the ICOH’s SC on MSDs and other interested scientists and members of the public recently discussed the scientific and clinical future of prevention of (work-related) MSDs during five round-table sessions at two ICOH conferences, in Cape Town, South Africa, in 2009 and in Angers, France, in 2010. The focus of the sessions was to discuss new developments that had taken place since 1996 with regard to the measures and classification systems used in research and practice, and agree on what is needed in the near future.

The discussion focused on three questions: At what degree of severity does musculoskeletal ill health, and do health problems related to MSDs, in an individual worker or in a group of workers justify preventive action in occupational health? What reliable and valid instruments do we have in research to distinguish ‘normal musculoskeletal symptoms’ from ‘serious musculoskeletal symptoms’ in workers? What measures or classification system of musculoskeletal health will we need in the near future to address musculoskeletal health and related work ability?

Forty-five minutes were allocated to each question. Three to four members of the SC were asked to prepare their views in advance, to start the discussion with expert opinions from different parts of the world. Immediately following the discussions, some statements were prepared by the organizers of the round-table discussions (J.S., M.H., F.V., R.B.) and discussed in a fourth round-table session. Four new, agreed-upon statements were extrapolated from the discussions and are given below.

1. *Musculoskeletal discomfort that is at risk of worsening with work activities, and that affects work ability or quality of life, needs to be identified.*Unpleasant sensations from the musculoskeletal system are experienced by everyone and can be adaptive in circumstances when muscle soreness is experienced after physical training, for example. In prevention of work-related MSDs, we need to assess musculoskeletal symptoms that have a potential of affecting workers’ health in a negative way. Symptoms at risk of worsening (e.g. paraesthesia as a first phase before pain may be present in entrapment syndromes) which reduce work ability or impair quality of life should be targeted. Questions like ‘What probabilities should be avoided?’ are likewise relevant. The core outcome domains recommended by the Initiative on Methods, Measurement, and Pain Assessment in Clinical Trials (IMMPACT) [16] for clinical trials of chronic pain can provide some guidance. These are: pain, physical functioning, emotional functioning, global wellbeing, symptoms and adverse events, and participant disposition. Pain that is worsening with work activities should be detected since this will probably influence productivity in addition to affecting quality of life. One idea that emerged involves a measure that couples pain with a measure of functioning. Examples of reliable and valid instruments used today to target part of these aspects are: the Work Ability Index (WAI; http://www.ttl.fi) [17], the Work Limitation Questionnaire (WLQ) [18] and the Work Role Functioning Questionnaire (WRFQ) [19]. Furthermore, the Nordic Musculoskeletal Questionnaire (NMQ) [20,21] and the Disabilities of the Arm, Shoulder and Hand (DASH) questionnaire [22] aim specifically at musculoskeletal symptoms and functioning.

2. *We need to know our options of actions before identifying workers at risk (providing evidence-based medicine and applying the principle of best practice).*Identifying workers at risk for developing MSDs or worsening of MSD symptoms related to work involves ethical issues. A management plan on how to give feedback to the worker and management (as well as unions and other stakeholders) should be considered before starting any health screening or surveillance of workers. The ICOH ethical guidelines state that we need to know our options of actions before identifying workers at risk. It is important to make some judgement about which symptoms are related to work exposures in order to predict what will get worse with work exposures and what will get better if work exposure is decreased.Action on individual resources or work demands should follow screening, using reliable and valid exposure instruments [6]. Criteria for addressing symptoms can be derived from knowledge of the prognosis, and of the effect of the symptoms on productivity and quality of life. It may be too early to recommend specific criteria or cut-off levels to identify workers at risk. However, examples from practices from different countries in setting criteria at the individual level that was asked for and mentioned in our discussion are: using a visual analogue scale (VAS) to assess pain and setting the criteria to 50 mm to identify ‘severe’ complaints/disorders. In Finland a rating of 70–100 is regarded as justifying intervention [23]. In France, a score of ≥50 is seen to indicate serious symptoms. Another example of action taken is when musculoskeletal complaints are the main health problem in workers scoring <6 for poor work ability on the 11-point scale of the Finnish WAI [24]; sometimes only the first item of the WAI is used and action can be taken based on that score [25]. In the Netherlands, an example of another type of health complaint, fatigue, was mentioned at the group level when the organizational or departmental criterion for action to be taken was defined as more than half of the workers scoring above the cut-off score on the Need for Recovery (NFR) after work scale [26].

3. *Classification systems and measures must include aspects such as the severity, frequency, and intensity of pain, as well as measures of impairment of functioning, which can help in prevention, treatment and prognosis.*To date, several classification systems for MSDs have been proposed and published in the literature, mainly aimed at defining diagnostic criteria. This is probably due to the importance of diagnosis for understanding the underlying pathological process as a prerequisite for the management of prevention and treatment of diseases.Even when the pathogenesis of illnesses is unclear, a case definition can be considered a useful way of classifying cases so that illnesses that share the same causes or a similar prognosis and response to treatment can be managed or prevented more effectively [27]. This links to the first statement “Musculoskeletal discomfort that is at risk of worsening with work activities, and that affects work ability or quality of life, needs to be identified”. The case definition for a disorder may vary according to the purpose for which it is being applied. Even broad, i.e. non-specific, case definitions may usefully identify workers at risk of progressing to more serious outcomes (see, e.g., [28].However, it is well recognized that it may not be sufficient to merely explore physical symptoms when nothing is known about the impact of the symptoms on functioning or work ability. The International Classification of Functioning (ICF) is an example of a classification system addressing functioning, disability and health in individuals and groups of individuals (http://www.who.int/classifications/icf/en/).One of the challenges for the scientific community that was agreed upon is to gain better understanding of the effect of the different aspects of the work environment on the functioning of the worker with certain MSD problems. We will then be able to propose better solutions to address these problems in time.

4. *We need to be aware of economic and/or socio-cultural consequences of classification systems and measures.*The scientific community should be aware of the societal impact of communicated work-related health problems. Legal disputes over compensation may affect work ability [29]. In Australia repetitive strain injuries (RSI) was debated in society during the 1980s which may have hampered adequate prevention at the time [30]. Reaction to musculoskeletal trauma may be influenced also by ethnicity [31]. We know from practised systems of defining occupational diseases that estimates of incidences may differ 60-fold between the different European Union (EU) countries. The consequences of classification systems and measures need to be elucidated and evaluated to minimize the risk of adverse effects on the individual worker and on society.

## Discussion

More attention in research should be directed to the functional impact of complaints in working life and also to solutions (interventions) to decrease this impact. There is a need for knowledge on what screening criteria best fit what type of interventions. Furthermore, with non-preventable factors, such as age, or where MSDs have are chronic, the goal is to reduce the impact of the condition on workers’ quality of life and ability to work. In fact, musculoskeletal function is related to physical capacity, one of the factors affecting the balance between work requirements and the worker’s performance capacity. In other words, musculoskeletal function is one of the determinants of work ability and may influence workers’ quality of life, the number of working days lost, and productivity.

As a consequence, musculoskeletal function is a parameter to consider in case definitions. It can be measured through physical examination and on the basis of specific protocols, with the aim to assess both basic functional abilities and work-specific abilities. New knowledge on how musculoskeletal function can be used in case definitions is urgent.

Tools to measure individuals’ perceived ability have also been developed in the form of self-administered questionnaires. The scores or indexes obtained are used to describe and monitor changes in function over time. However, information is needed on minimal detectable changes and minimal clinical significant changes of these instruments.

Given these objectives, there is a need for knowledge on how to integrate the diagnostic classification systems of MSDs with procedures to evaluate physical functioning and workers’ perceived disability. This will allow a deeper understanding of the impact of musculoskeletal conditions on work ability and it will enable us to assess the effectiveness of preventive strategies or interventions aimed at reducing the burden of MSDs.

The adversarial and acrimonious climate in some countries, due, in large part, to issues surrounding compensation, have hindered the development of scientific knowledge on the prevention of work-related MSDs. There is therefore a need for knowledge about how the scientific community can realistically communicate the impact of work-related MSDs on sustainable work ability to governmental bodies and society. Integrating health economics into future musculoskeletal research may be an effective way of getting through to the public, politicians companies and managers. We need new knowledge on the costs of preventable musculoskeletal disorders[32]. Finally we need more information on productivity and sustainable musculoskeletal health [33].

Future position papers of the ICOH’s SC on MSDs should focus on the following topics: relevant interventions when physical demands remain high; and implementation of interventions with focus on how to standardize and implement a more individualized approach.

## Conclusions

Four new, agreed-upon statements concerning prevention of musculoskeletal disorders in workers were extrapolated from the discussions with scientists in the field: 1. Musculoskeletal discomfort that is at risk of worsening with work activities, and that affects work ability or quality of life, needs to be identified. 2. We need to know our options of actions before identifying workers at risk (providing evidence-based medicine and applying the principle of best practice). 3. Classification systems and measures must include aspects such as the severity, frequency, and intensity of pain, as well as measures of impairment of functioning, which can help in prevention, treatment and prognosis. 4. We need to be aware of economic and/or socio-cultural consequences of classification systems and measures.

## Competing interest

The authors declare that they have no competing interests.

## Authors’ contributions

M.H., J.S., F.V. and R.B. organized the round-table discussions which led to formulation of the statements. M.H. and J.S. drafted the manuscript. A.D., B.E., J.G., F.V. and R.B. contributed to and discussed the manuscript. All readers have read and approved the manuscript.

## Author information

M.H. is Professor in Occupational Medicine and chief physician at the Occupational and Environmental Medicine Clinic at Sahlgrenska University Hospital in Gothenburg. Together with the late professor Åsa Kilbom, he chaired the first Prevention of Work-Related Musculoskeletal Disorders (PREMUS) conference in Stockholm in 1992. He participated in drafting the first position paper for the ICOH’s SC on MSDs.

F.V. is Professor in Occupational Medicine at the University of Bologna and Director of the Occupational Health Unit, Sant'Orsola Malpighi Hospital, Bologna, Italy

R.B. is Researcher in Occupational Medicine at the University of Bologna and occupational physician at the Occupational Health Unit, Sant'Orsola Malpighi Hospital, Bologna, Italy

A.D. is an associate professor in occupational medicine and epidemiology in U1018, Inserm “Population-Based Epidémiological Cohorts” Research Platform in Versailles -St Quentin University and Occupational Health Department unit, Poincaré Teaching Hospital (Paris hospital), Garches, France

J.G. is an assistant professor of epidemiology in the Department of Public Health at Temple University, Philadelphia, Pennsylvania, USA

B.E. is Professor in Occupational Medicine, Industrial and Environmental Medicine, Chief of Division of General Medical Science and Director of Institute of Clinical and Translational Sciences, at Washington University in St-Louis, Missouri, USA

J.S. JS is an associate professor in occupational medicine and occupational health and is principal investigator at the Coronel Institute of Occupational Health, Academic Medical Center, University of Amsterdam, Amsterdam, The Netherlands.

## Pre-publication history

The pre-publication history for this paper can be accessed here:

http://www.biomedcentral.com/1471-2474/13/109/prepub
